# Multiple sources, shared readouts: the attribution gap in circular RNA immunotherapy for esophageal squamous cell carcinoma

**DOI:** 10.3389/fimmu.2026.1985510

**Published:** 2026-10-21

**Authors:** Zhenhua Li, Jiawen Wang, Huilai Lv, Fan Zhang, Bokang Sun, Chao Huang, Zhao Liu, Ziqiang Tian

**Affiliations:** Department of Thoracic Surgery, The Fourth Hospital of Hebei Medical University, Shijiazhuang, China

**Keywords:** circular RNA, effect attribution, esophageal squamous cell carcinoma, immunogenicity, innate RNA sensing, PKR, tumor immunotherapy, type I interferon

## Abstract

Therapeutic circular RNAs (circRNAs) are progressing from preclinical development to clinical evaluation, and esophageal squamous cell carcinoma (ESCC) may become a future indication for circRNA-based immunotherapy, potentially including first-line use. However, three focal sources—the intrinsic immunogenicity of exogenous circRNAs, the abundance of the endogenous circRNA pool, and patients’ baseline interferon states—can act through different mechanisms but converge on the same readouts, including interferon output, protein yield, and immune phenotypes. Their individual contributions are therefore difficult to distinguish, complicating efforts to establish dose–response relationships. This Perspective examines this structural barrier to effect attribution and proposes strategies at three levels: product characterization and control, effect dissection, and baseline adjustment. We argue that studies of circRNA immunotherapy in ESCC and related settings should incorporate concurrent measurement of these three key, measurable sources at the design stage, with causal inference complementing association analyses to grade the reliability of attribution. Such an approach could help reduce attribution bias and provide a foundation for subsequent dose optimization and risk stratification.

## 1 Introduction

Therapeutic circRNAs are advancing rapidly in oncology, immunology, and rare diseases (1), while therapeutic vaccines for solid tumors have entered phase I clinical testing (2). Although ESCC is not yet an indication for therapeutic circRNAs, circRNAs are implicated in key processes such as immune evasion and tumor progression in this disease (3, 4). Immunotherapy has meanwhile become a major treatment modality, incorporated into first-line regimens (5) and increasingly evaluated in the neoadjuvant setting (6, 7). Accordingly, therapeutic circRNA approaches may warrant future clinical investigation in ESCC and could ultimately be considered for first-line use if supported by future evidence.

Current studies focus primarily on the regulatory roles of circRNAs in tumor progression and metastasis, with less attention to the immune effects associated with circRNA therapy (8, 9): once delivered into the cytoplasm, therapeutic circRNAs can engage innate RNA-sensing pathways (10), with downstream effects captured by readouts such as interferon output, protein yield (i.e., translational output or expression of the encoded protein), and immune phenotypes (11–13). These readouts, however, are not specific to exogenous RNA; the endogenous circRNA pool within tumors and the patient’s baseline immune state can also shape them. Although the lipid components of delivery carriers can activate innate immune responses independently of RNA (14), and concomitant chemotherapy can also affect the relevant readouts (15), these effects can be separated using appropriate controls (16), thereby reducing their impact on subsequent studies. By contrast, the intrinsic immunogenicity of exogenous circRNA (12, 17), the abundance of the endogenous circRNA pool (18), and the patient’s baseline interferon state (19) are difficult to disentangle using simple controls, yet are directly relevant to establishing dose–response relationships and therefore warrant closer consideration. Moreover, current best-practice standards for circRNA research focus mainly on molecular identity, quantification, and functional validation (20, 21). Because these standards were not developed specifically for measurement design in therapeutic studies, they do not require concurrent recording of exogenous circRNA properties, the endogenous circRNA pool, and the patient’s baseline interferon state. Consequently, overlapping readouts can obscure the contribution of each source, creating a structural problem of effect attribution.

We therefore argue that studies of therapeutic circRNAs in ESCC immunotherapy should establish standards for concurrently recording and evaluating indicators of exogenous and endogenous circRNAs together with patients’ baseline immune status. When supported by appropriate study designs and prespecified statistical methods, these measurements may enable approximate estimation of source-specific contributions, helping to clarify dose–response relationships and inform treatment risk stratification. This attribution problem is not unique to ESCC. We use ESCC as an example because neoadjuvant immunotherapy provides an opportunity for paired sampling before and after treatment (6, 7), facilitating concurrent observation of these three sources (**Figure 1**).

**Figure 1:**
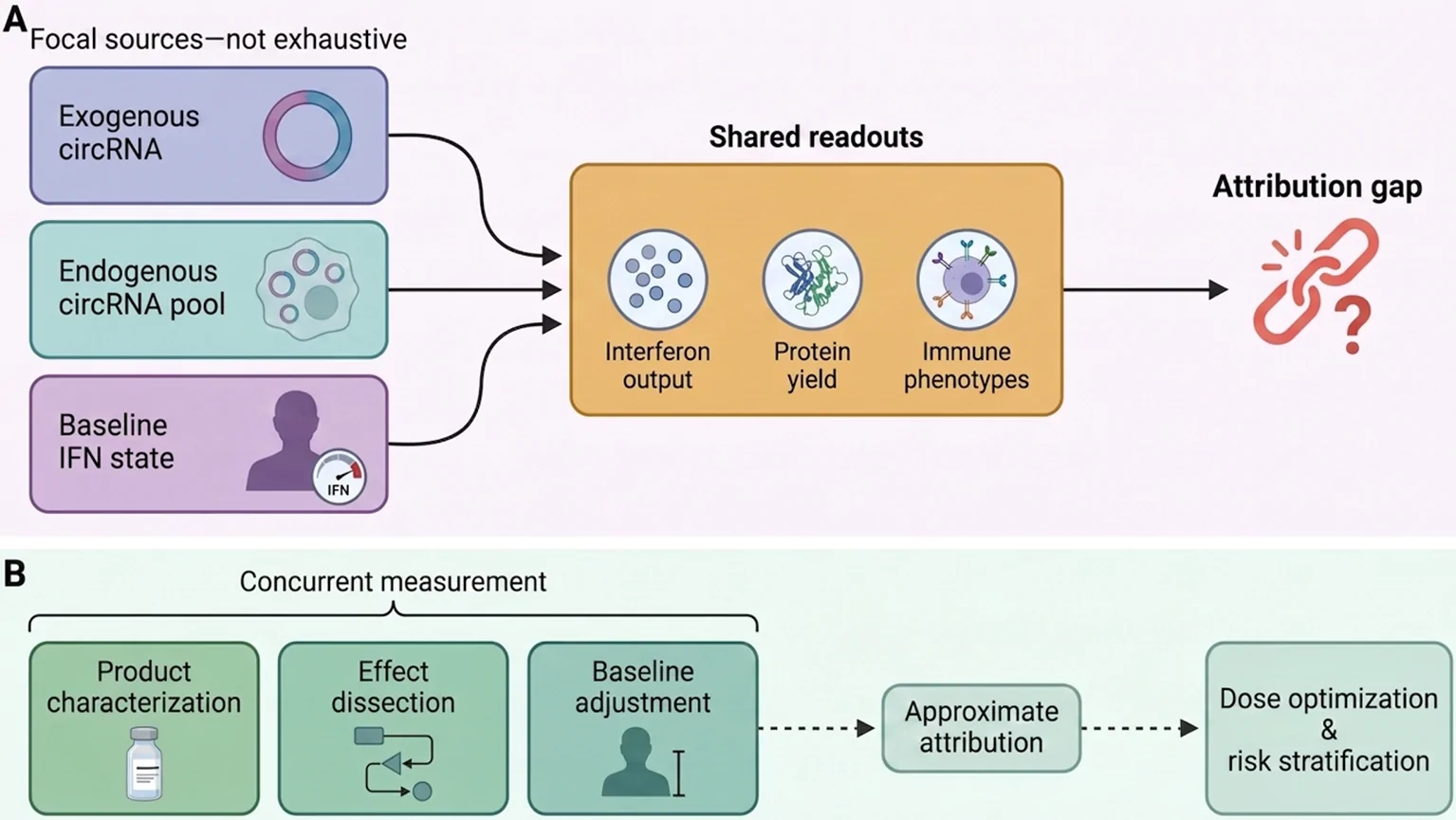
Prospective measurement framework for multisource effect attribution in circular RNA immunotherapy studies for esophageal squamous cell carcinoma. **(A)** Three focal, measurable, but non-exhaustive sources—the intrinsic immunogenicity of exogenous therapeutic circular RNA (circRNA), the endogenous circRNA pool, and the patient’s baseline interferon (IFN) state (exogenous circRNA is the primary source, the endogenous pool is a background determinant, and the baseline interferon state acts as a modifier)—can each influence shared readouts, including interferon output, protein yield, and immune phenotypes. Because these readouts are not source-specific, observed changes cannot be confidently attributed to the therapeutic product on the basis of these readouts alone, creating an attribution gap. **(B)** Concurrent product characterization, experimental effect dissection, and baseline adjustment may reduce attribution bias and, when combined with appropriate study designs and prespecified statistical methods, support approximate estimation of source-specific contributions. This framework may improve the interpretation of dose–response relationships and provide a basis for subsequent dose optimization and risk stratification, but it does not establish patient-level causal contributions. Dashed arrows denote proposed relationships that require prospective validation. Created in BioRender. wang, J. (2027) https://BioRender.com/gj6fu5e.

## 2 Structural barriers to effect attribution

Among the factors that can shape these readouts, this Perspective focuses on three key and measurable sources: the intrinsic immunogenicity of exogenous therapeutic circRNAs, the abundance of the endogenous circRNA pool, and the patient’s baseline interferon state. Each has a distinct mechanistic basis, yet their effects converge on the same set of readouts, making their relative contributions difficult to distinguish.

For exogenous circRNAs, immunogenicity is associated mainly with molecular identity, preparation purity, and manufacturing residues. Studies have shown that exogenous circRNAs generated using nonhuman bacteriophage introns can activate RIG-I and induce type I interferon (17), whereas N^6^-methyladenosine (m^6^A)-modified circRNAs can attenuate or evade this response through YTH domain-containing family protein 2 (YTHDF2)-mediated sequestration (22). The effects of exogenous circRNAs on interferon expression therefore depend in part on their provenance and molecular identity. Preparation purity also matters. When purification is inadequate, residual double-stranded RNA (dsRNA), 5′-triphosphate termini of uncircularized RNA, and hydrolytic fragments—even at low levels—can elicit robust immune responses. Under the tested conditions, extensively purified circRNAs have been reported to avoid RIG-I and TLR sensing, attenuating these responses while improving the stability of expression of the encoded protein (12, 23, 24). Immunogenicity may also be affected by sequences retained during manufacturing. For example, self-splicing intron-based circularization can leave td- or *Anabaena*-derived fragments embedded in the mature circle; these sequences may alter local three-dimensional folding, enhance immunogenicity, activate immune recognition, and elicit innate immune responses (25). Together, these factors can produce a variable and complex immunogenicity profile. Even among exogenous circRNAs, differences in manufacturing and construct design may lead to marked differences in expression of the encoded protein: residual linear contaminants can trigger immune responses and reduce the stability of expression of the encoded protein (12), whereas optimized constructs can sustain antigen translation with limited PKR activation (13). Thus, the direction of changes in shared readouts may be difficult to predict even when only exogenous factors are considered, making it difficult to infer, much less quantify, the intrinsic immunogenicity of a circRNA from those readouts alone.

The endogenous circRNA pool further complicates interpretation of these readouts. Cellular circRNAs can contain 16–26-bp imperfect duplex structures that bind to PKR and inhibit its activation. When poly(I:C) or viral stimulation activates the OAS–RNase L pathway, widespread degradation of circRNAs can relieve this inhibition and permit PKR activation (18). This general mechanism may be relevant to ESCC, although it has not been directly established in this disease. In ESCC, circRNA expression profiles differ markedly between tumors and adjacent tissues and involve numerous, widely distributed molecules (26). These observations make modulation of PKR by endogenous circRNAs plausible, but it remains a mechanistic hypothesis requiring direct validation. Because the abundance of the circRNA pool is tissue-specific, the strength of its regulation of PKR is unlikely to be constant. Furthermore, circFAM120B can promote ubiquitination-dependent degradation of PKR in ESCC, while circFAM120B itself is often expressed at low levels in tumor tissue and plasma, with variation among patients (4). Together, these findings suggest that PKR and its downstream readouts may already be shaped by endogenous factors before exposure to exogenous circRNA. Post-treatment changes therefore should not automatically be attributed to the exogenous agent; endogenous influences must also be considered, further complicating efforts to quantify the respective contributions of endogenous and exogenous factors to changes in expression of the encoded protein.

The patient’s baseline interferon state can also shape both the amplification and branching of the sensing network and therefore should not be overlooked. In tumor cells under chronic interferon stimulation, network components may shift in opposing directions: increased expression of RIG-I, MDA5, and PKR can heighten sensitivity to dsRNA (19), whereas USP18 can attenuate IFNAR signaling and limit the response magnitude in this context (27). Moreover, immune and stromal composition varies markedly among patients with ESCC (28), making it plausible that the same dose could produce substantially different, or even opposing, effects across baseline contexts. This possibility underscores the potential importance of baseline state. Yet a randomized first-line trial of PD-1-based combination regimens that improved outcomes in ESCC did not use interferon subtyping as a stratification framework (5). This absence alone does not establish long-standing neglect or quantify the extent of baseline confounding. It does, however, illustrate that baseline interferon data are not routinely used to separate baseline contributions from treatment-induced effects, further complicating attribution.

Ultimately, mechanisms arising from multiple sources converge on the same set of readouts. Their respective contributions are difficult to distinguish and quantify, and interpretation becomes even more challenging when a single marker has context-dependent meanings. PD-L1, for example, may reflect adaptive immune resistance in an IFN-γ-rich environment, constitutive expression driven by oncogenic signaling (29), or changes in protein stability caused by deubiquitination (30). The same readout can therefore represent distinct biological processes. In ESCC, circNF1 further illustrates this ambiguity because it can upregulate PD-L1 through both transcriptional induction and enhanced protein stability, and its upregulation is closely associated with response to anti-PD-L1 therapy (3). Thus, an increase in PD-L1 after dosing may reflect adaptive resistance in an IFN-γ-rich environment or may be related to endogenous circNF1 levels; attribution is difficult on the basis of this readout alone. This problem is not simply an oversight in study execution; it reflects a structural gap in measurement design that must be addressed prospectively.

Recent findings provide further support for this concern. For exogenous circRNA, the route of administration can alter the nature of the immune response even when the construct is unchanged: intravenous injection favors systemic CD8^+^ T-cell activation, whereas intradermal injection favors antitumor immunity (31). This indicates that exogenous immune signals are also modulated by factors such as the administration route. For endogenous circRNA, circAP2B1 and circVEGFC, which are upregulated in ESCC, can promote M2 macrophage polarization through distinct mechanisms (32, 33), indicating that endogenous circRNAs can independently act on the same immune axis. Regarding baseline state, upregulation of IFN-γ-response genes in CD8^+^ effector T cells and NK cells before neoadjuvant immunochemotherapy in patients with ESCC is closely associated with pathological response (6). This further indicates that baseline immune heterogeneity can influence response interpretation independently of treatment. These complexities both impede attribution to a specific circRNA and increase the difficulty of attribution, underscoring the urgent need to address this problem.

## 3 Discussion

Recent work in the field has addressed several aspects of exogenous circRNA, including platform engineering, purification and quality control, delivery, and mechanistic immune analyses (1). However, two other core factors—the state of the endogenous circRNA pool and the patient’s baseline immune state—remain largely absent from current measurement designs. Randomization alone cannot resolve the resulting problem of mechanistic effect attribution by source, although addressing this problem is an important prerequisite and methodological safeguard for rigorous circRNA immunotherapy research. Among these three factors, exogenous circRNA is the primary source, the endogenous pool is a background determinant, and the baseline interferon state acts as a modifier. We therefore call for balanced attention to exogenous therapeutic circRNA, the endogenous circRNA pool, and the patient’s baseline immune state as three focal, measurable sources, while recognizing that they do not constitute an exhaustive inventory of contributors to the shared readouts. Corresponding strategies include product characterization and control through systematic annotation and closed-loop validation, effect dissection through three-arm controls combined with blockade–rescue experiments, and baseline adjustment through interferon profiling combined with longitudinal sampling. Together, these approaches may reduce multisource interference in effect attribution, allow approximate estimation of source-specific contributions, and provide a conceptual framework for dose optimization and risk stratification in future circRNA immunotherapy studies in ESCC.

To address incomplete information on the therapeutic product itself, best-practice standards for circRNA research (20, 21) could guide systematic batch-level documentation of process parameters, including the circularization method, impurity profile, nucleoside modifications, and delivery-vector composition. Back-splice-junction sequencing, poly(A)-tailing assays (34), and purity analysis by reversed-phase high-performance liquid chromatography (HPLC) (35) could then provide orthogonal confirmation that the product is circular and quantify residual linear RNA. Together, these measures would help trace unexpected immunogenicity to specific manufacturing steps and establish a closed quality-control loop linking product documentation to manufacturing verification.

To disentangle effects that converge on the same readouts, the three-arm control design used in lipid nanoparticle (LNP)–mRNA platforms—empty carrier, noncoding RNA, and coding RNA—could be adapted to circRNA systems, including ESCC models, to interrogate the immune contribution of each component (16). IFNAR blockade could help isolate interferon-dependent effects (16), whereas knockdown followed by reconstitution of PKR and RNase L could establish a blockade–rescue sequence (18) to test, within experimental systems, whether endogenous and exogenous RNA species perturb one another during therapeutic exposure and whether observed effects depend causally on specific pathways. These perturbations should be paired with measurements of p-IRF3 and IFN-β transcription; p-PKR, p-eIF2α, and translational output; expression of the encoded protein; maturation of antigen-presenting cells; antigen-specific CD8^+^ T-cell responses (13, 36); and inhibitory feedback such as PD-L1 (3, 29). In addition, empty carriers should be matched as closely as possible to the loaded particles in key biophysical parameters, including particle size, surface charge, and apparent pK_a_, to reduce interference from carrier differences. Together, component controls and pathway-resolved readouts could support effect dissection, reduce indiscriminate attribution of multisource effects to the encoded product, and enable approximate estimation and initial quantification of component-level contributions within preclinical experimental systems. They would not, by themselves, quantify patient-level source contributions.

To address patient-to-patient baseline variation in response to the same dose, paired pre- and post-treatment sampling strategies used in neoadjuvant immunochemotherapy for ESCC (6, 7) could be adapted. Peripheral blood could be collected before dosing, early after dosing (6–24 h), at an intermediate interval (3–7 days), and at imaging assessment; tissue biopsies could be obtained at appropriate time points only when clinically feasible and ethically justified. These temporal windows could help distinguish pre-existing baseline differences from treatment-induced changes. Immune-cell composition inferred from pretreatment peripheral blood mononuclear cell (PBMC) profiles and interferon-related transcriptional features could also be explored for risk stratification of immune-related adverse events, but this strategy remains exploratory and requires validation in an independent ESCC cohort (37). Additional measurements should include type I interferon pathway markers (ligands, IFNAR, and pSTAT), negative-feedback markers (USP18 and SOCS) (27, 38), IFN-γ-related T-cell-inflamed signatures, stress markers such as PKR and eIF2α, and spatial immune composition. Such multidimensional characterization of the baseline immune state could help distinguish interferon signals arising from different sources and support more reliable assessment of dose–response relationships.

Although changes in readouts can indicate pathway status to some extent, they provide associational evidence and do not establish causal relationships. Causal inference should therefore complement association analyses for source attribution (39), allowing the reliability of attribution to be graded in specific contexts. In preclinical experimental systems, sources can be directly perturbed, with between-group differences from three-arm controls, perturbations of endogenous-pool abundance, and blockade–reconstitution experiments serving as the principal evaluation measures. Comparisons across source-specific groups with comparable baselines can then estimate exogenous effects, endogenous effects, and pathway dependence separately, providing relatively high reliability of attribution. Clinical studies require a different approach. The endogenous circRNA pool and baseline interferon state cannot be randomly assigned in patients, yet they can act jointly with exogenous factors on the same pathway to regulate its activity. Pathway dependence can therefore correspond to multiple sources, making source attribution difficult. Appropriate variables should be selected using causal diagrams (40) and measured and adjusted for before dosing to reduce heterogeneity and standardize comparisons as far as possible. Prespecified subgroup analyses and interaction tests can then indirectly assess how dose effects vary with endogenous-pool abundance and baseline interferon state. Although the reliability of this indirect approach is limited, agreement between findings from perturbations in experimental models and paired patient sampling can substantially improve the reliability of attribution (41–43).

## 4 Conclusion

Recent studies have highlighted limitations in purity testing and immune assessment of exogenous circRNA, but the circRNA therapeutics field has yet to fully recognize the problem of effects from multiple sources converging on the same readouts. In particular, the state of the endogenous circRNA pool and the patient’s baseline immune state have not been systematically incorporated into measurement designs. We therefore call for balanced attention to the immunogenicity of exogenous circRNA, the abundance of the endogenous circRNA pool, and the patient’s baseline interferon state. These three focal, measurable sources—without implying that they are exhaustive—should be assessed concurrently in studies of circRNA immunotherapy for ESCC and related settings, with causal inference complementing association analyses. Such designs may reduce effect-attribution error and misinterpretation of dose–response relationships, thereby providing a basis for subsequent risk stratification.

## Data Availability

The original contributions presented in the study are included in the article/supplementary material, further inquiries can be directed to the corresponding author/s.
